# Traditional Chinese medicine therapy decreases the pneumonia risk in patients with dementia

**DOI:** 10.1097/MD.0000000000004917

**Published:** 2016-09-16

**Authors:** Shun-Ku Lin, Yueh-Ting Tsai, Pei-Chia Lo, Jung-Nien Lai

**Affiliations:** a Department of Chinese Medicine, Taipei City Hospital, Renai Branch; b Institute of Traditional Medicine, School of Medicine, National Yang-Ming University, Taipei; c School of Chinese Medicine, College of Chinese Medicine, China Medical University; d Departments of Chinese Medicine, China Medical University Hospital, Taichung Taiwan.

**Keywords:** dementia, Ma-Xing-Gan-Shi-Tang, pneumonia, the national health insurance research database, traditional Chinese medicine, Yin-Qiao-San

## Abstract

Pneumonia is a frequent complication in dementia patients and is associated with high mortality rates. The aim of this retrospective cohort study was to determine whether traditional Chinese medicine (TCM) therapy can decrease pneumonia risk in dementia patients. The cohort dataset was obtained from the Longitudinal Health Insurance Database 2005, a sublibrary of the National Health Insurance Research Database, containing all medical data of 1 million beneficiaries, randomly selected from the all Insurers in year 2005.

Newly diagnosed dementia patients (n = 9712) without pneumonia were analyzed from January 1997 to December 2003. After matching by sex, age, urban level, Charlson comorbidity index, insured amount, and comorbidities, 1376 pairs (1:1) of TCM and non-TCM users were acquired. Every dementia patient was individually recorded from 1997 to 2012 to identify pneumonia incidence (onset after 3 months of dementia diagnosis).

Demographic characteristics, Charlson comorbidity index, comorbidities, behavioral and psychological symptoms of dementia, and psychotropic drugs were also investigated. Cox proportional regression was used to compute hazard ratios and 95% confidence intervals (CIs) after adjustment for the above-mentioned variables.

There were 419 (30.5%) and 762 (55.4%) pneumonia cases in the TCM and non-TCM cohorts during a mean follow-up period of 7.6 years. The adjusted hazard ratios (95% CI) for pneumonia admission was 0.62 (0.55–0.70) for the TCM group.

Patients who received TCM therapy at higher cumulative doses or for longer periods experienced increased protection from pneumonia admission. Ma-Xing-Gan-Shi-Tang, Yin-Qiao-San, and Xiao-Qing-Long-Tang might represent possible formulae reducing the incidence of pneumonia. TCM might be associated with a lower risk of pneumonia in dementia patients.

## Abstract 2

Supplemental Digital Content is available in the text

## 1 Introduction

Pneumonia is a frequent complication in dementia patients and is associated with high mortality rates.^[1,2]^ Of interest, angiotensin-converting enzyme inhibitors have been found to lower the risk of pneumonia and decrease mortality related to pneumonia.^[3]^ Further, vaccination,^[4]^ correct posture,^[5]^and swallowing rehabilitation^[6]^ have also been suggested to reduce the incidence of pneumonia. In addition, a prospective, observer-blinded, randomized controlled trial revealed Ban-Xia-Houpu-Tang (HangeKobokuTo in Japanese, English Name: Pinellia and Officinal Magnolia Bark Decoction) can reduce the incidence of aspiration pneumonia,^[7]^ and follow-up studies confirmed it might improve the swallowing reflex in patients with dementia.^[8]^ Further, an observational study found combined using traditional and western medicine might decrease the hospital stay length, body temperature, and white blood cell count compared with western medicine treatment only.^[9]^

However, there is currently no nationwide study focused on the effects of traditional Chinese medicine (TCM) on pneumonia prevention in dementia patients, and no study has yet screened for potential effective formulae. With this in mind, the aims of the study were to determine whether TCM therapy could decrease pneumonia admission risk in people with dementia and to identify Chinese medicine formulae which might early treat pulmonary symptoms and protect the patient not to progress into pneumonia admission.

## 2 Materials and methods

### 2.1 Data sources

The presented research was a retrospective cohort study which was given official approval by the Institutional Review Board of Taipei City Hospital, Taiwan (Case Number TCHIRB-1020816-E). The cohort was selected from the National Health Insurance Research Database (NHIRD), a nationwide database containing more than 97% of the people in Taiwan. The NHIRD includes clinical and demographic information such as sex, birth date, outpatient visits, and admission diagnoses according to the International Classification of Diseases, Ninth Revision (ICD-9) classification, Clinical Modification.^[10]^ Further, all prescription drugs (involving name, dosage, and duration) and medical procedures (including classification, time, and cost) are also recorded in detail.^[11,12]^

The Longitudinal Health Insurance Database 2005 is a sublibrary of NHIRD, including all clinical medical data of 1,000,000 beneficiaries from January 1, 1996 to December 31, 2012, randomly selected from all beneficiaries of the NHIRD in 2005. The distribution of demographic factors was similar between the Longitudinal Health Insurance Database 2005 and the entire population in Taiwan, as reported by the National Health Research Institutes.^[13]^ Taiwan National Institutes of Health collects insurance claims data into NHIRD. All Taiwanese medical researchers are allowed to apply NHIRD, National Institutes of Health would review the IRB approval and research projects researchers.

### 2.2 Study sample

**Figure 1:**
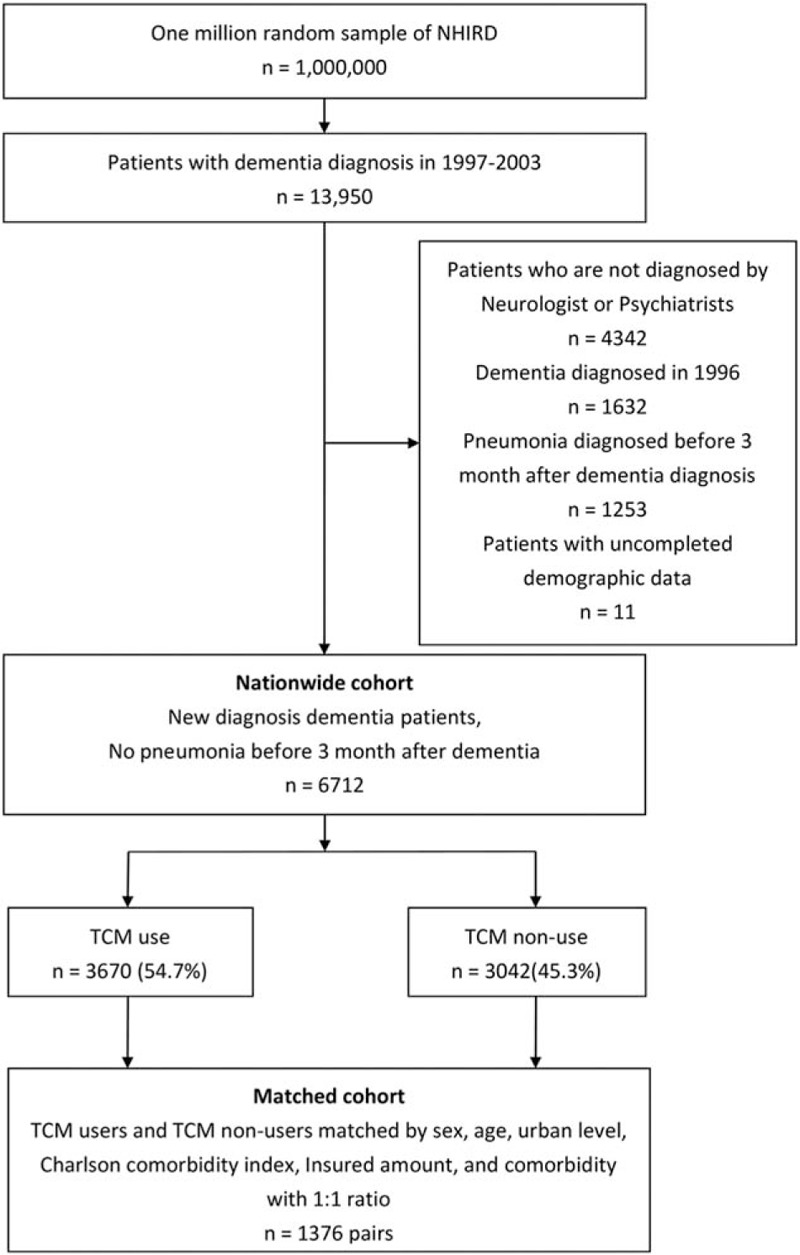
Flowchart of subject recruitment from the 1-million random sample of the National Health Insurance Research Database from 1997 to 2003 in Taiwan. There were 2752 dementia patients (1376 pairs of traditional Chinese medicine [TCM] and non-TCM matching) were included in this prospective cohort study.

As seen in Fig. 1, we selected a representative nationwide cohort (n = 6712) from these datasets, in which all participants were newly diagnosed with dementia (ICD-9 codes 290, 294, 331.0) by a neurologist or psychiatrist, from the 1 million individuals included between 1997 and 2003. Patients with any history of pneumonia within the 1st 3 months after the dementia diagnosis (n = 1253) or with incomplete demographic data (n = 11) were excluded.

### 2.3 Exposure and follow-up

To examine the effect of TCM treatment on pneumonia admission, we divided the study patients into TCM users (n = 3670) and non-TCM users (n = 3042). The TCM users included all participants who had been taking TCM between the 1st dementia diagnosis and the pneumonia admission date (or the end of the December 2012 for those who were in the nonpneumonia group). The non-TCM cohort consisted of patients who had not taken any TCM. Both cohorts were matched regarding age category in 2003, sex, insured amount category, urbanization level, Charlson comorbidity index, and the frequencies of certain comorbid diseases (diabetes mellitus, cerebral vascular incident, heart failure, Parkinson disease, chronic obstructive pulmonary disease, and tuberculosis) at a ratio of 1:1. Overall, 2752 patients (1376 pairs) were included in this prospective analysis. We included all patients who received TCM treatment, regardless of how many days of use. However, different doses and treatment time may affect the outcome. Therefore, we divided the patient population by the cumulative time (<200days, 200–399 days, and ≥400 days) and the cumulative dose (<1000 g, 1000–1999 g, and ≥2000 g) of TCM use.

This investigation was planned to involve all pneumonia admissions until the December 31, 2012, unless the participants had died. Exposure began at the 1st date of TCM visit or dementia diagnosis (in the non-TCM group), and the end point was defined as the date of pneumonia admission or the last medical record before the December 31, 2012. We collected the names of all TCM herbs or formulae, daily dose, frequency, duration of use, and dates of the prescriptions. The length and dose of TCM treatment exposure were measured as the cumulative days and daily dose until the end of the follow-up, respectively.

### 2.4 Definition of pneumonia

The major outcome of the presented study was pneumonia admission. All pneumonia admission cases were selected by the ICD-9 codes 480, 481, 482, 483, 484, 485, 486, and 507. In Taiwan, pneumonia admission is fully covered by National Health Insurance, but the medical records need to be investigated by a panel of experts. We define 1st hospitalized with pneumonia as outcome research, track time starts at dementia diagnosis, and terminates on the date of the 1st hospitalization for pneumonia. We do not include the 2nd and subsequent hospitalization for pneumonia in the study. Tracheal intubation or intensive care unit requirements during hospitalization were recorded as minor outcomes.

### 2.5 Potential confounders

We systematically identified any potential confounders for pneumonia, including the following comorbidity diagnoses recorded during the follow-up: diabetes mellitus, cerebral vascular incident, heart failure, Parkinson disease, chronic obstructive pulmonary disease, tuberculosis, gastro-esophageal reflux disease, chronic kidney disease, epilepsy, coronary heart disease, asthma, liver cirrhosis, and cancer.^[14]^ Moreover, we considered the behavioral and psychological symptoms of dementia (BPSD; delirium, delusions, depression, behavioral disturbance, sleep disturbances, and hallucination) and the Charlson comorbidity index in the modeling.^[15]^ The comorbidities, BPSD, and Charlson comorbidity index were identified according to the ICD-9 codes, listed in Appendix 1. Socio-demographic characteristics, including age at the time of dementia diagnosis, sex, insured amount, and urbanization level, were also taken into account in the analyses.

The insured amount, which was calculated from the household income, was divided into 4 strata (dependent, 1–19,999, 20,000–39,999, and above 40,000 New Taiwan Dollars per month, equal to less than 666, 666–1333, more than 1334 USD), with dependent policyholders defined as people without salaries. The urbanization levels were classified into 4 categories (very high, high, moderate, and low).^[16]^ The urbanization level is a good indicator to investigate the development stratification of Taiwan townships; index calculation urbanization level has included the variables included: population density, educational levels, population ratio of elder people, agriculture workers ratio, and the number of physicians per 100,000 people.

According to previous studies, the use of psychiatric medication might affect the incidence of pneumonia admission.^[17,18]^ Therefore, all psychotropic drugs, including antimanic, antidepressant, anti-Alzheimer, anxiolytic, and antipsychotic drugs prescribed by neurologists and psychiatrists, as shown in Appendix 2, were analyzed. These drugs are fully reimbursed by the NHI and cannot be prescribed without a doctor's order.

### 2.6 Statistical analysis

To ensure the matching was appropriate, we analyzed the differences in the demographic factors, the Charlson comorbidity index, comorbidities, and psychotropic drugs using the Chi-square test. The incidence of pneumonia admission was calculated during the follow-up period. We used the Kaplan–Meier method to create survival curves of pneumonia, and we used log-rank test to examine the differences in pneumonia admission risks between TCM users and nonusers. Cox proportional regression models were created to calculate the hazard ratios and the accompanying 95% confidence intervals (CIs) after adjustment for the possible confounders. We use the 2-tailed test and assumed a significance level of *P*-values <0.05. All analyses were carried out using SAS statistical software (version 9.4; SAS Institute Inc., Cary, NC).

Further, the risks of pneumonia admission between different TCM use duration and dose strata were also tested to examine possible dose-effect relations. To determine potential effect confounders, we stratified the subgroups by the presence of comorbidities, demographics factors, BPSD, and psychological medication in the TCM use cohort. To survey the potential TCM formulae that might decrease the risk of pneumonia admission in dementia patients, we analyzed all individual formulae used by dementia patients. We also used the Cochran-Armitage test to examine the trend between different strata. All Chinese herbal products covered by the Bureau of National Health Insurance are registered with the Department of Chinese Medicine and Pharmacy, the competent authority of TCM, and are manufactured according to good manufacturing practice standards. Chinese herbal products created using the same formulations were considered equal.

## 3 Results

The mean age of the study participants was 78.6 ± 10.5 years, and 50.4% were female. The mean exposure time to TCM was 6.7 ± 1.8 years, and the cumulative dosage was 1365.8 ± 397.2 g. Table 1 shows the distributions of the demographic characteristics and medical conditions in the matched cohort. Higher proportions of diseases (gastro-esophageal reflux disease, chronic kidney disease, epilepsy, coronary heart disease, asthma, liver cirrhosis, and cancer), BPSD (delirium, delusions, depression, sleep disturbances, and hallucination), and medication (z-drug, benzodiazepines, atypical antipsychotics, antimanic drug, selective serotonin reuptake inhibitor, tricyclic antidepressant, and norepinephrine reuptake inhibitor) were found in the TCM groups.

**Table 1:**
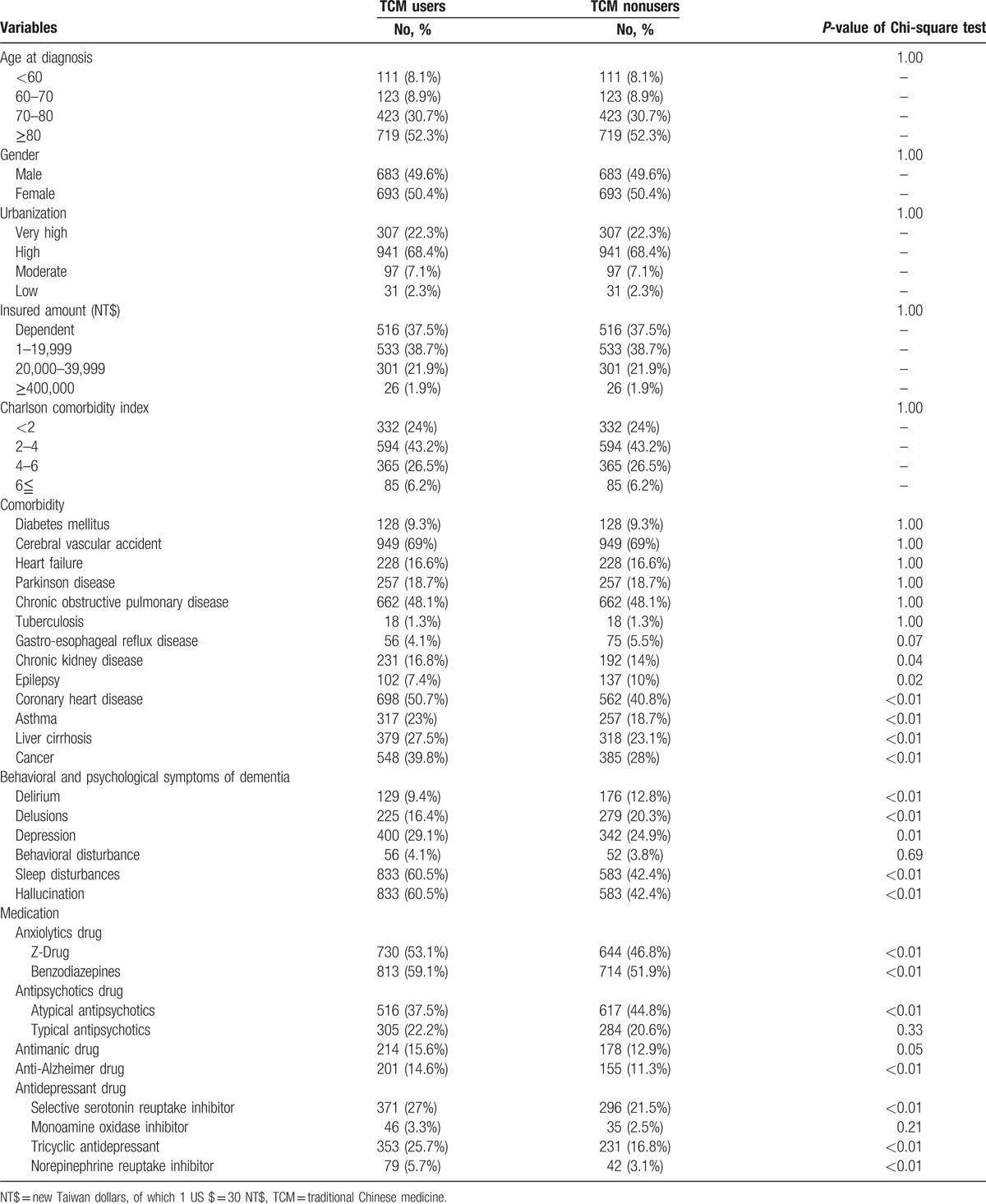
Demographic and medical characteristics of the matched dementia cohort.

A total of 1376 TCM users were included in the study, of whom 419 (30.5%, incidence density 3.5%) were admitted for pneumonia; in contrast, in the non-TCM group (n = 1376), 762 (55.4%, incidence density 7.4%) were admitted with pneumonia (adjusted hazard ratio 0.62, 95% CI 0.55–0.70) during the follow-up. Further, the dementia TCM users suffering from pneumonia (average 7.0 years with standard deviation 3.3 years) was later than non-TCM users (average 4.7 years with standard deviation 3.2 years).

**Figure 2:**
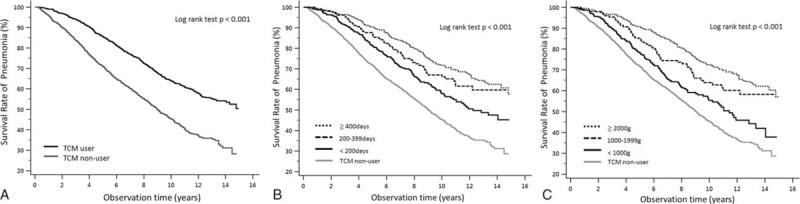
(A) Survival curve of hospitalized pneumonia in patients with dementia. Kaplan–Meier survival curves and log-rank analyses revealed significant differences in the rates of pneumonia admission between traditional Chinese medicine (TCM) and non-TCM users (log-rank test, *P* < 0.001). (B) Survival curve of hospitalized pneumonia in patients with dementia according to traditional Chinese medicine time stratification. TCM usage for <200, 200–399, and ≧400 days resulted in significantly reduced rates of pneumonia admission (log-rank test, *P* < 0.001). (C) Survival curve of hospitalized pneumonia in patients with dementia according to traditional Chinese medicine dose stratification. Patients with cumulative doses of <1000 g, 1000–1999, and ≧2000 g showed significantly reduced rates of pneumonia admission compared to patients without TCM use (log-rank test, *P* < 0.001).

For patients receiving TCMs for <200, 200–399, and ≧400 days, the adjusted hazard ratios were 0.66 (95% CI 0.57–0.77), 0.53 (95% CI 0.40–0.71), and 0.47 (95% CI 0.39–0.58), respectively (Table 2). As seen in Fig. 2A, Kaplan–Meier survival curves and log-rank analyses revealed significant differences in the rates of pneumonia admission between TCM and non-TCM users (log-rank test, *P* < 0.001). Moreover, the analyses revealed significant differences in the pneumonia admission incidence among the subgroups of TCM users. TCM usage for <200, 200–399, and ≧400 days resulted in significantly reduced rates of pneumonia admission (log-rank test, *P* < 0.001; Fig. 2A), and patients with cumulative doses of <1000 g, 1000–1999, and ≧2000 g also showed significant differences compared to patients without TCM use (hazard ratio 0.68 [95% CI 0.58–0.81], 0.59 [95% CI 0.45–0.75], and 0.50 [95% CI 0.42–0.59]; log-rank test, *P* < 0.001; Table 2 and Fig. 2C). All subgroups in the TCM use group showed protective effects against pneumonia requiring intensive care. In TCM groups, there were 52.0% patients that received tracheal intubation treatment and 62.1% required intensive care from a total of 419 patients. On the other hand, only 46.2% and 47.2% patients needed tracheal intubation and intensive care in the non-TCM groups of 762 patients in sum. The TCM group had a higher risk of both tracheal intubation use (hazard ratio 1.50 [95% CI 1.04–2.16]) and intensive care unit (1.39 [1.09–1.77]) following pneumonia admission (Table 3).

**Table 2:**
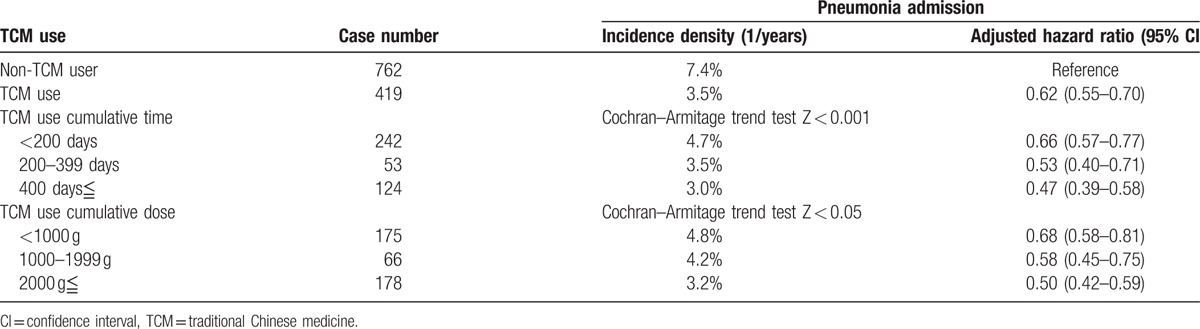
Multivariable Cox model measured hazard ratios and 95% CIs of TCM for pneumonia in matched cohort.

**Table 3:**
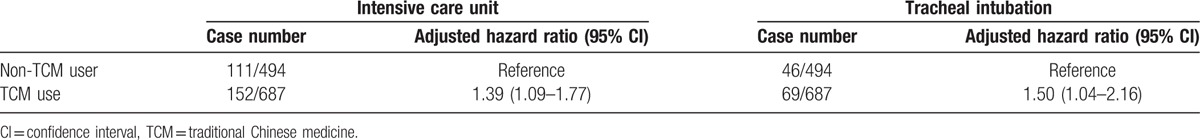
Multivariable Cox model measured hazard ratios and 95% CIs of TCM for tracheal intubation use and intensive care unit after pneumonia admission.

To understand the effects of various socio-demographic and medical confounders, we analyzed stratified subgroups in the nationwide TCM use cohort of dementia patients (Table 4). Patients who were older, low urbanization level, male, and high Charlson comorbidity index were at higher risk of pneumonia admission. In view of BPSD, delusions, depression, and sleep disturbances demonstrated higher adjusted hazard ratios for pneumonia admission (1.65 [95% CI 1.40–1.94], 1.16 [95% CI 1.01–1.34], and 1.35 [95% CI 1.54–1.19], respectively). In addition, cerebral vascular incident, heart failure, Parkinson disease, tuberculosis, and chronic obstructive pulmonary disease were associated with high risks of pneumonia admission. Benzodiazepine was associated with a higher adjusted hazard ratio (1.15, 95% CI 1.01–1.32) of pneumonia, whereas tricyclic antidepressant users showed a reduced risk (0.92, 95% CI 0.88–0.97).

**Table 4:**
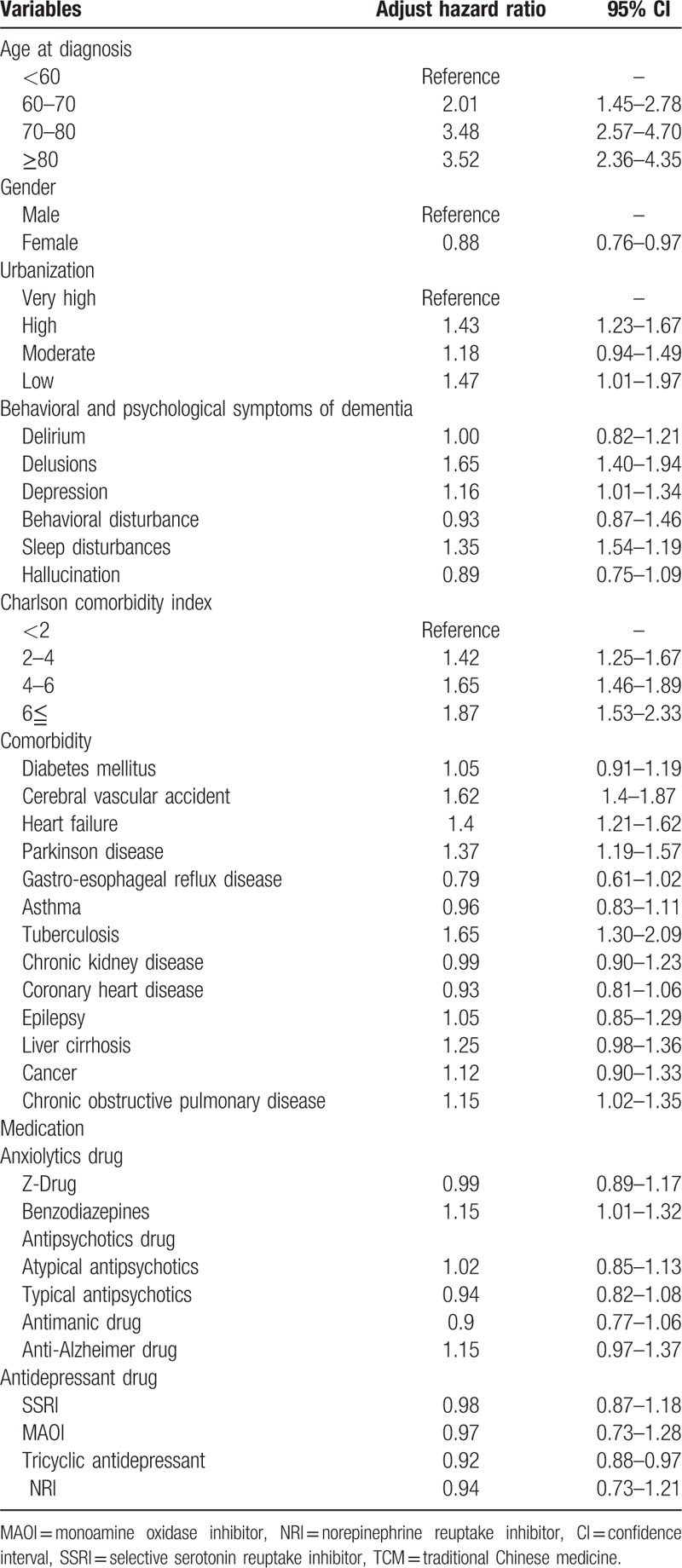
Hazard ratios of pneumonia risk in nationwide TCM use cohort of dementia patients, analyzed by multivariable cox proportional hazards regression model and 95% CIs.

Finally, among the commonly used Chinese medicine formulae, Ma-Xing-Gan-Shi-Tang (Ephedra, Apricot Kernel, Licorice, and Gypsum Decoction) had the lowest adjusted hazard ratio (0.64, 95% CI 0.52–0.77), as determined by Cox proportional hazards regression models after adjustment. Yin-Qiao-San (Lonicera and Forsythia Powder), Xiao-Qing-Long-Tang (Minor Green-Blue Dragon Decoction), Ban-Xia-Hou-Po-Tangs (Pinellia and Officinal Magnolia Bark Decoction), and Xin-Yi-Qing-Fei-Tang (Magnolia Flower Lung-Clearing Decoction) also showed significant protective effects on pneumonia admission, as shown in Table 5.

**Table 5:**
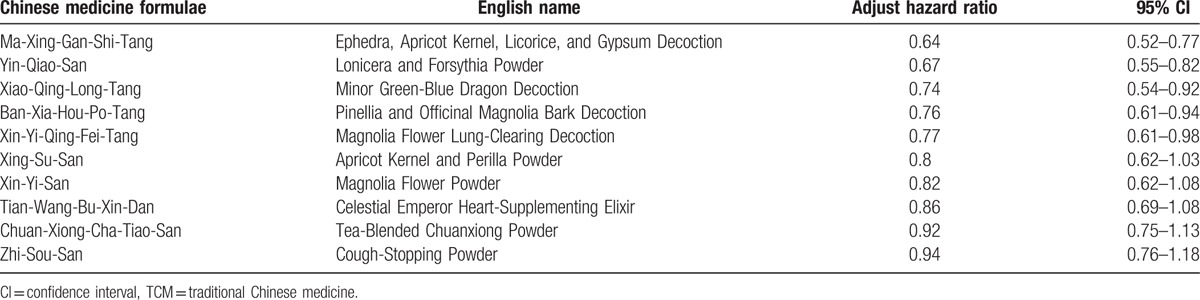
Adjusted Cox proportional hazards ratio of the commonly used Chinese medicine formulae in national TCM cohort.

## 4 Discussion

In this retrospective population-based cohort study, the results suggested TCM treatment was associated with a lower incident density and hazard ratio of pneumonia admission in dementia patients. Further, we analyzed TCM effects related to exposure-duration and dosage on the pneumonia admission, and also tested the effects of different TCM formulae. Factors statistically significantly associated with the incremental risk of pneumonia admission were male gender, age 60 and above, low urbanization, benzodiazepine use, high score of Charlson comorbidity index, previous diagnosis of cerebral vascular incident, heart failure, Parkinson disease, tuberculosis, and chronic obstructive pulmonary disease.

Our previous study found dementia patients with hypertension, cerebral vascular incident, diabetes mellitus, insomnia, depression, abnormal behavior, and hallucinations had greater demand for receiving TCM and TCM formulae, and these were widely used to relieve the behavioral and psychological symptoms.^[19]^ In addition, another study confirmed TCM healthcare as a safer alternative to reduce the use of hypnotic drugs,^[20]^ which are potentially hazardous for the dementia population. Directly via relieving BSPD or indirectly via reducing the severity of comorbidities or sedative-hypnotic dependence, the present study demonstrated TCM healthcare was associated with a significant 0.38-fold reduced risk of pneumonia admission compared with non-TCM users.

The cumulative time and dosage of TCM exposure were found to influence the protective effects against pneumonia admission. Although the duration of less than 200 days showed a statistically significant lower risk compared to nonuse, this effect was more obvious after at least 400 days of TCM prescription. The present results also show the hazard ratio of pneumonia admission was reduced from 0.66 to 0.47 in patients who had received TCM for more than 400 days. The cumulative dosage showed a similar performance. For patients who had received more than 6000 g of TCM, the risk was reduced from 0.68 to 0.50 compared to patients who had received less than 3000 g (Fig. 2).

To our limited knowledge, this research is the 1st nationwide study to assess the effect of TCM on the incidence of pneumonia admission. Of these patients with dementia, the hospital admission rate for pneumonia is 5.5%. Although the incidence of pneumonia admission appears to be low compared with the estimates of previous surveys,^[21,22]^ all pneumonia admissions were diagnosed by board-certified physicians where the possibility of either selection or recall bias can be excluded. Among dementia patients with severe pneumonia, the rates of tracheal intubation and intensive care were 48.3 and 52.5%, respectively. Nearly half of pneumonia patients required endotracheal intubation or intensive care, raising the need for early detection and treatment of respiratory infection. Research on optimal treatment and long-term outcomes of survival and mortality rate are warranted. Although, the duration from dementia diagnosis to pneumonia admission in TCM group was longer than in the non-TCM group, the TCM group had a higher proportion of both tracheal intubation use and intensive care unit following pneumonia admission. This might indicate TCM healthcare decreased the risk of pneumonia admission, but could not slow the progress of the pneumonia after admission.

In a previous nationwide study, most dementia patients using TCM also used western medicine.^[19]^ Hence, to investigate the effect of combination therapy, we created stratified subgroups from the national TCM cohort (Table 5) for those with or without using concomitant psychotropic drugs. We observed TCM users who used benzodiazepine were associated with an increased risk of pneumonia admission. In a previous study, benzodiazepines were associated with an increased risk and mortality of community-acquired pneumonia,^[23]^ and have been reported to might induce delirium in hospitalizations.^[24]^ On the other hand, tricyclic antidepressants were linked to lower risk in TCM user groups, while a prospectively study suspected large tricyclic antidepressant ingestion might induce pulmonary edema and aspiration pneumonia,^[25]^ no significant relation between tricyclic antidepressant use and mortality risk in pneumonia patients was found.^[26]^ Little is known regarding the potential interactions of Chinese herbal medicine with hypnotic drugs among the dementia population; therefore, clinicians and public-health policy analysts should focus on the potential long-term impact of herb-drug concurrence use. Moreover, older age, low urbanization, cerebral vascular incident, heart failure, Parkinson disease, delusions, and depression were also associated with a high risk of pneumonia admission, similar to in a previous retrospective clinical database study.^[27]^

In the presented study, Ma-Xing-Gan-Shi-Tang (Ephedra, Apricot Kernel, Licorice, and Gypsum Decoction), Yin-Qiao-San (Lonicera and Forsythia Powder), Xiao-Qing-Long-Tang (Minor Green-Blue Dragon Decoction), Ban-Xia-Hou-Po-Tangs (Pinellia and Officinal Magnolia Bark Decoction), and Xin-Yi-Qing-Fei-Tang (Magnolia Flower Lung-Clearing Decoction) were found to show significantly lower hazard ratios for pneumonia admission compared to nonuse. In a previous observer-blinded, randomized, controlled trial, a significantly reduced aspiration pneumonia risk was observed after Ban-Xia-Hou-Po-Tangs (Pinellia and Officinal Magnolia Bark Decoction) treatment, with an adjusted hazard ratio slightly lower than that in the present study;^[28]^ Ma-Xing-Gan-Shi-Tang (Ephedra, Apricot Kernel, Licorice, and Gypsum Decoction) was reported to relieve the symptoms of chronic bronchitis^[29]^ and lower respiratory tract infection.^[30]^ It was indicated to protect influenza virus infection through inhibiting both viral RNA and protein synthesis,^[31]^ and could reduce lung inflammation and inhibit virus replication in mice infected with respiratory syncytial virus.^[32]^ A systematic review article also showed Ma-Xing-Gan-Shi-Tang combined with western medicine could improve the clinical symptoms of pneumonia.^[33]^ Further, Ma-Xing-Gan-Shi-Tang (Ephedra, Apricot Kernel, Licorice, and Gypsum Decoction) combined with Yin-Qiao-San (Lonicera and Forsythia Powder) has been found to reduce fever and symptoms in patients with H1N1 influenza virus infection.^[34]^ Besides, Yin-Qiao-San (Lonicera and Forsythia Powder) was explored to prevent drug-induced pulmonary fibrosis in a mice model and was found to significantly improve health-related quality of life in a randomized, double-blind placebo-controlled trial.^[35]^

Xiao-Qing-Long-Tang (Minor Green-Blue Dragon Decoction) is a commonly prescribed Chinese herbal products for adult-onset asthma in Taiwan^[36]^ and has indicated enhanced protection of pneumonia vaccination through augmenting the nasal antiviral IgA antibody and serum antiviral IgG antibodies.^[37,38]^ It was also found to effect against human respiratory syncytial virus infection by inhibiting viral attachment, internalization, and syncytial formation.^[39]^ In accordance with these results, a randomized controlled multicenter clinical trial showed TCM treatment could also enhance the quality of life among community-acquired pneumonia patients, and the mortality rate was similar to that of patients treated with antiinfection agents plus conventional medicine.^[40]^

In our study, we found that the TCM formulae which could prevent pneumonia admission are used to treat respiratory tract infections. We infer the probable cause is the use of the formulae may be the early treatment of respiratory diseases to block disease progression and avoid severe pneumonia. We recommend that dementia patients who with upper respiratory infection should receive Chinese medical treatment as soon as possible to prevent the subsequent deterioration, rather than long-term use after the diagnosis of dementia.

Although our research indicates that some of the TCM formulae can reduce the risk of hospitalization for pneumonia, we still have to remind the possible toxicity of TCM and herb–drug interaction. For example, the ephedra included in Ma-Xing-Gan-Shi-Tang and Xiao-Qing-Long-Tang has the effect of stimulating sympathetic nervous system increasing cardiovascular and cerebrovascular disease risk.^[42,43]^ Also, Zizyphi spinosi semen contained in the Tian-Wang-Bu-Xin-Dan would influence GABA_A_ receptor and enhance the sedative effect of benzodiazepine,^[44]^ which was the most common psychotropic drug which combined use with TCM.^[20]^

## 5 Limitation

There are some potential limitations of this study worth mentioning. First, the diagnoses (including dementia, pneumonia, and comorbidities) were obtained only from NHIRD and were coded using the ICD-9 classification, Clinical Modification system. The Bureau of National Health Insurance inspects all diagnoses through a very strict peer-review process and refuses inappropriate diagnoses. This process helps to ensure the accuracy of the diagnoses but may cause under-estimating of disease.^[21,22]^ This might be a possible explanation for the pneumonia admission incidence being lower compared with previous epidemiological studies.^[41]^ Another limitation is the fact some potentially confounders, such as tobacco use, alcohol intake, blood pressure, blood sugar level, body mass index, level of education, and baseline cardiopulmonary function, were not included in our database. In addition, the national insurance system paid only Chinese medicine powder form, different herbal products types including decoction, extract, pill, home remedies, or herbal dietary supplements were not covered; therefore, we might have underestimated the use of TCM use. Furthermore, we could not exclude the influence of selection bias from the research findings. The low frequency of admission treatment in TCM group might be related to the patent's or physician's preference but not relevant to the preventative effect of herbal medicine. Patients in the TCM group could have a tendency not to participate early in the admission treatment for pneumonia which resulted in the high proportion of intensive care unit treatment. In the follow of the medical ethics in clinical practice, such selection bias is based on the respect to patient's autonomy. The NHI system allowed all patients choice medical therapy freely, and the selection bias is unavoidable in the NHIRD cohort study. We use the matching method to reduce selection bias, and we found the dose-dependent and time-dependent response of the decreasing risk for pneumonia admission. Finally, the NHIRD includes only the Taiwanese insurer, and the results of the presented study might therefore not be applicable to populations in other countries.

## 6 Conclusion

TCM may have a protective effect on pneumonia admission in dementia patients. Longer exposure and a larger cumulative dosage were associated with greater protective effects against pneumonia. Ma-Xing-Gan-Shi-Tang (Ephedra, Apricot Kernel, Licorice, and Gypsum Decoction), Yin-Qiao-San (Lonicera and Forsythia Powder), and Xiao-Qing-Long-Tang (Minor Green-Blue Dragon Decoction) showed the highest protective effects of the TCM formulae. Further mechanistic research and clinical trials are needed to evaluate the precise effects of Chinese medicine formulae on the risk of pneumonia.

## Supplementary Material

Supplemental Digital Content

## References

[1] Mitchell SL Teno JM Kiely DK The clinical course of advanced dementia. *N Engl J Med* 2009; 15:1529–1538. 19828530 10.1056/NEJMoa0902234 PMC2778850

[2] Zuliani G Galvani M Sioulis F Discharge diagnosis and comorbidity profile in hospitalized older patients with dementia. *Int J Geriatr Psychiatry* 2012; 27:313–320. 21538539 10.1002/gps.2722

[3] Arai T Sekizawa K Ohrui T ACE inhibitors and protection against pneumonia in elderly patients with stroke. *Neurology* 2005; 64:573–574. 15699404 10.1212/01.WNL.0000150897.14961.0F

[4] Nichol KL Wuorenma J von Sternberg T Benefits of influenza vaccination for low-, intermediate-, and high-risk senior citizens. *Arch Intern Med* 1998; 158:1769–1776. 9738606 10.1001/archinte.158.16.1769

[5] Terré R Mearin F Effectiveness of chin-down posture to prevent tracheal aspiration in dysphagia secondary to acquired brain injury. A videofluoroscopy study. *Neurogastroenterol Motil* 2012; 24:414–419. 22309385 10.1111/j.1365-2982.2011.01869.x

[6] Troche MS Okun MS Rosenbek JC Aspiration and swallowing in Parkinson disease and rehabilitation with EMST: a randomized trial. *Neurology* 2010; 75:1912–1919. 21098406 10.1212/WNL.0b013e3181fef115 PMC2995389

[7] Iwasaki K Kato S Monma Y A pilot study of banxia houpu tang, a traditional Chinese medicine, for reducing pneumonia risk in older adults with dementia. *J Am Geriatr Soc* 2007; 55:2035–2040. 17944889 10.1111/j.1532-5415.2007.01448.x

[8] Iwasaki K Cyong JC Kitada S A traditional Chinese herbal medicine, banxia houpo tang, improves cough reflex of patients with aspiration pneumonia. *J Am Geriatr Soc* 2002; 50:1751–1752. 12366640 10.1046/j.1532-5415.2002.50479.x

[9] Li Y Tian WM Wu Q Combined traditional and western medicine in the treatment of 100 elderly patients suffering from severe pneumonia: an analysis of clinical results. *Zhongguo Wei Zhong Bing Ji Jiu Yi Xue* 2011; 23:44–45. 21251367

[10] Centers for Disease Control and Prevention. International Classification of Diseases, Ninth Revision (ICD-9) (online). Available at: http://www.cdc.gov/nchs/icd/icd9.htm [Accessed January 1, 2016.].

[11] Chi NF Chien LN Ku HL Alzheimer disease and risk of stroke: a population-based cohort study. *Neurology* 2013; 80:705–711. 23303851 10.1212/WNL.0b013e31828250af

[12] Chien IC Lin YC Chou YJ Treated prevalence and incidence of dementia among National Health Insurance enrollees in Taiwan, 1996–2003. *J Geriatr Psychiatry Neurol* 2008; 21:142–148. 18474723 10.1177/0891988708316859

[13] National Health Research Institutes. National Health Insurance Research Database (online). Available from URL: http://nhird.nhri.org.tw/date_01.htm [Accessed January 1, 2016].

[14] Torres A Peetermans WE Viegi G Risk factors for community-acquired pneumonia in adults in Europe: a literature review. *Thorax* 2013; 68:1057–1065. 24130229 10.1136/thoraxjnl-2013-204282 PMC3812874

[15] Charlson ME Pompei P Ales KL A new method of classifying prognostic comorbidity in longitudinal studies: development and validation. *J Chronic Dis* 1987; 40:373–383. 3558716 10.1016/0021-9681(87)90171-8

[16] Liu CY Hung YT Chuang YL Incorporating development stratification of Taiwan townships into sampling design of large scale health interview survey. *J Health Management* 2006; 4:1–22.

[17] Pratt N Roughead EE Salter A Choice of observational study design impacts on measurement of antipsychotic risks in the elderly: a systematic review. *BMC Med Res Methodol* 2012; 12:72. 22682666 10.1186/1471-2288-12-72 PMC3447663

[18] Trifiró G Sultana J Spina E Are the safety profiles of antipsychotic drugs used in dementia the same? An updated review of observational studies. *Drug Saf* 2014; 37:501–520. 24859163 10.1007/s40264-014-0170-y

[19] Lin SK Tsai YT Lai JN Demographic and medication characteristics of traditional Chinese medicine users among dementia patients in Taiwan: A nationwide database study. *J Ethnopharmacol* 2015; 161:108–115. 25527314 10.1016/j.jep.2014.12.015

[20] Lee KH Tsai YT Lai JN Concurrent use of hypnotic drugs and Chinese herbal medicine therapies among Taiwanese adults with insomnia symptoms: a population-based study. *Evid Based Complement Alternat Med* 2013; 2013:987862. 24204397 10.1155/2013/987862 PMC3800591

[21] Fillit H Geldmacher DS Welter RT Optimizing coding and reimbursement to improve management of Alzheimer's disease and related dementias. *J Am Geriatr Soc* 2002; 50:1871–1878. 12410910 10.1046/j.1532-5415.2002.50519.x

[22] Taylor DH Jr Fillenbaum GG Ezell ME The accuracy of medicare claims data in identifying Alzheimer's disease. *J Clin Epidemiol* 2002; 55:929–937. 12393082 10.1016/s0895-4356(02)00452-3

[23] Obiora E Hubbard R Sanders RD The impact of benzodiazepines on occurrence of pneumonia and mortality from pneumonia: a nested case-control and survival analysis in a population-based cohort. *Thorax* 2013; 68:163–170. 23220867 10.1136/thoraxjnl-2012-202374

[24] Lin RY Heacock LC Fogel JF Drug-induced, dementia-associated and non-dementia, non-drug delirium hospitalizations in the United States, 1998-2005: an analysis of the national inpatient sample. *Drugs Aging* 2010; 27:51–61. 20030432 10.2165/11531060-000000000-00000

[25] Shannon M Lovejoy FH Jr Pulmonary consequences of severe tricyclic antidepressant ingestion. *J Toxicol Clin Toxicol* 1987; 25:443–461. 2894471 10.3109/15563658708992648

[26] Barnett MJ Perry PJ Alexander B Risk of mortality associated with antipsychotic and other neuropsychiatric drugs in pneumonia patients. *J Clin Psychopharmacol* 2006; 26:182–187. 16633149 10.1097/01.jcp.0000203598.43314.34

[27] Hak E Bont J Hoes AW Prognostic factors for serious morbidity and mortality from community-acquired lower respiratory tract infections among the elderly in primary care. *Fam Pract* 2005; 22:375–380. 15805127 10.1093/fampra/cmi020

[28] Iwasaki K Kato S Monma Y A pilot study of banxia houpu tang, a traditional Chinese medicine, for reducing pneumonia risk in older adults with dementia. *J Am Geriatr Soc* 2007; 55:2035–2040. 17944889 10.1111/j.1532-5415.2007.01448.x

[29] Zhu Y Liu X Treatment of chronic bronchitis with modified ma xing shi gan tang and er chen tang. *J Tradit Chin Med* 2004; 24:12–13. 15119160

[30] Yue Q Ni ZY Effect of ma-xin-shi-gan tang on the immune function in children with acute lower respiratory tract infection. *Zhong Xi Yi Jie He Za Zhi* 1990; 10:581–583. 2268918

[31] Hsieh CF Lo CW Liu CH Mechanism by which ma-xing-shi-gan-tang inhibits the entry of influenza virus. *J Ethnopharmacol* 2012; 143:57–67. 22710290 10.1016/j.jep.2012.05.061

[32] Chen ZG Luo H Wang SC Antiviral effects of Jinxin oral liquid against respiratory syncytial virus infection in the BALB/c mice model. *J Ethnopharmacol* 2015; 162:287–295. 25593018 10.1016/j.jep.2015.01.002

[33] Li L Lu FG He QH Efficacy of Maxing Shigan Decoction combined with western medicine for pneumonia in children: a systematic review and meta-analysis. *Zhong Xi Yi Jie He Xue Bao* 2009; 7:809–813. 19747433 10.3736/jcim20090902

[34] Wang C Cao B Liu QQ Oseltamivir compared with the Chinese traditional therapy maxingshigan-yinqiaosan in the treatment of H1N1 influenza: a randomized trial. *Ann Intern Med* 2011; 155:217–225. 21844547 10.7326/0003-4819-155-4-201108160-00005

[35] Wong W Lam CL Fong DY Treatment effectiveness of two Chinese herbal medicine formulae in upper respiratory tract infections – a randomized double-blind placebo-controlled trial. *Fam Pract* 2012; 29:643–652. 22490614 10.1093/fampra/cms027

[36] Wang HM Lin SK Yeh CH Prescription pattern of Chinese herbal products for adult-onset asthma in Taiwan: a population-based study. *Ann Allergy Asthma Immunol* 2014; 112:465–470. 24656660 10.1016/j.anai.2014.02.012

[37] Nagai T Yamada H In vivo anti-influenza virus activity of Kampo (Japanese herbal) medicine “sho-seiryu-to” – stimulation of mucosal immune system and effect on allergic pulmonary inflammation model mice. *Immunopharmacol Immunotoxicol* 1998; 20:267–281. 9653672 10.3109/08923979809038544

[38] Nagai T Urata M Yamada H In vivo anti-influenza virus activity of Kampo (Japanese herbal) medicine “Sho-seiryu-to” – effects on aged mice, against subtypes of a viruses and B virus, and therapeutic effect. *Immunopharmacol Immunotoxicol* 1996; 18:193–208. 8771367 10.3109/08923979609052732

[39] Chang JS Yeh CF Wang KC Xiao-Qing-Long-Tang (Sho-seiryu-to) inhibited cytopathic effect of human respiratory syncytial virus in cell lines of human respiratory tract. *J Ethnopharmacol* 2013; 147:481–487. 23542148 10.1016/j.jep.2013.03.044

[40] Li J Yu X Li S Randomized controlled multicenter clinical trial for integrated treatment of community-acquired pneumonia based on traditional Chinese medicine syndrome differentiation. *J Tradit Chin Med* 2012; 32:554–560. 23427388 10.1016/s0254-6272(13)60070-9

[41] Bauer K Schwarzkopf L Graessel E A claims data-based comparison of comorbidity in individuals with and without dementia. *BMC Geriatr* 2014; 28:10–14. 24472217 10.1186/1471-2318-14-10 PMC3909381

[42] Yao Y Zhang X Wang Z Deciphering the combination principles of Traditional Chinese Medicine from a systems pharmacology perspective based on Ma-huang Decoction. *J Ethnopharmacol* 2013; 150:619–638. 24064232 10.1016/j.jep.2013.09.018

[43] Kim EJ Chen Y Huang JQ Evidence-based toxicity evaluation and scheduling of Chinese herbal medicines. *J Ethnopharmacol* 2013; 146:40–61. 23286904 10.1016/j.jep.2012.12.027

[44] Han H Ma Y Eun JS Anxiolytic-like effects of sanjoinine A isolated from Zizyphi Spinosi Semen: possible involvement of GABAergic transmission. *Pharmacol Biochem Behav* 2009; 92:206–213. 19101585 10.1016/j.pbb.2008.11.012

