# Supplementary material for: Traditional Chinese medicine therapy decreases the pneumonia risk in patients with dementia
Source: Medicine (Baltimore). 2016 Sep 16;95(37):e4917. doi: 10.1097/MD.0000000000004917 (PMC5402612; doi:10.1097/MD.0000000000004917)
Supplement: Supplemental Digital Content [file medi-95-e4917-s001.doc]

Appendix 1. International Classification of Disease, Ninth Revision (ICD-9) Codes of Co-morbidity Diseases Used in This Study

| **Diseases** | **ICD-9 Codes** |
| --- | --- |
| Dementia | 290, 294, 331.0 |
| Pneumonia | 480, 481, 482, 483, 484, 485, 486, 507 |
| **Comorbidity Diseases** | **ICD-9 Codes** |
| Diabetes mellitus | 249, 250 |
| Cerebral vascular accident | 430, 431, 432, 433, 434, 435, 436, 437, 438 |
| Heart failure | 428 |
| Parkinson disease | 332 |
| Chronic obstructive pulmonary disease | 490, 491, 492, 494, 495, 496 |
| Tuberculosis | 011, 012 |
| Gastro-esophageal reflux disease | 530.8 |
| Chronic kidney disease | 585, 586, 588 |
| Epilepsy | 345 |
| Coronary heart disease | 410, 411, 412, 413, 414 |
| Asthma | 493 |
| Liver cirrhosis | 571 |
| Cancer | 140 - 238 |
| **Behavioral and Psychological Symptoms** | **Icd-9 Codes** |
| Delirium | 290.11, 290.3, 290.41 |
| Delusions | 290.20, 290.12, 290.42 |
| Depression | 290.13, 290.21, 290.43 |
| Sleep disturbances | 780.5, 307.4 |
| Hallucination | 7801, 291.3, 292.8 |
| Behavioral disturbance | 294.11, 294.21 |

Appendix 2. Detail of Anti-Alzheimer and BPSD Drugs Used In This Study

| **Drug Classification** | **Detail** |
| --- | --- |
| **Anti-Alzheimer Drugs** | Deprenyl, Galantamine, Donepezil, Memantine, And Rivastigmine |
| **Antipsychotics** |  |
| Typical Antipsychotic | Chlorpromazine, Chlorprothixene, Thioridazine, Levomepromazine, Loxapine, Perphenazine, Trifluoperazine, Haloperidol, Fluphenazine, Droperidol, Zuclopenthixol, Flupentixol, Prochlorperazine |
| Atypical Antipsychotics | Amisulpride , Aripiprazole , Clozapine , Olanzapine , Paliperidone , Quetiapine, Risperidone, Sulpiride, Ziprasidone, Zotepine |
| **Antidepressant Drug** |  |
| Serotonin Antagonist And Reuptake Inhibitors(SSRI) | Citalopram , Paroxetine , Fluoxetine , Fluvoxamine , Sertraline , Trazodone |
| Norepinephrine Reuptake Inhibitors(NRIs) | Atomoxetine , Agomelatine , Mirtazapine , Bupropion |
| Tricyclic Antidepressants | Amitriptyline , Clomipramine , Doxepin , Imipramine |
| Monoamine Oxidase Inhibitor(MAOIs) | Moclobemide, Selegiline |
| **Anxiolytics Drug** |  |
| Benzodiazepine | Chlordiazepoxide , Clonazepam , Diazepam , Lorazepam , Oxazepam , Flunitrazepam, Nitrazepam, Diazepam, Nitrazepam |
| Z-Drugs | Zopiclone, Zaleplon, Zolipidem |
| **Antimanic Drug** | Carbamazepine , Divalproex, Lamotrigine , Lithium, Oxcarbazepine , Topiramate , Trazodone, Valproic_Acid |

**Appendix 3. Name, Unaccented Pinyin, and Ingredient Herbs** of Chinese Medicine Formulae

| Chinese Name | Unaccented Pinyin | English Name | Prescription Frequency | Ingredients |
| --- | --- | --- | --- | --- |
| 麻杏甘石湯 | Ma-Xing-Gan-Shi-Tang | Ephedra, Apricot Kernel, Licorice, and Gypsum Decoction | 1009 | Ma-Huang(Ephedrae Herba Cruda), Xing-Ren(Armeniacae Semen), Gan-Cao(Glycyrrhizae Radix cum Liquido Fricta), Shi-Gao(Gypsum Crudum) |
| 銀翹散 | Yin-Qiao-San | Lonicera and Forsythia Powder | 977 | Jin-yin-hua(lonicera [flower] Lonicerae Flos), Lian-Qiao(erect hypericum Hyperici Erecti Herba), Jing-Jie-Sui(schizonepeta spike Schizonepetae Flos), Dan-Dou-Chi(fermented soybean Sojae Semen Praeparatum), Jie-Geng (platycodon [root] Platycodonis Radix), Bo-He(mint Menthae Herba), Niu-Bang-Zi(arctium [seed] Arctii Fructus), Gan-Cao(Glycyrrhizae Radix cum Liquido Fricta), Zhu-Ye(bamboo leaf Lophatheri Folium), Wei-Gen(phragmites [root] Phragmitis Rhizoma) |
| 小青龍湯 | Xiao-Qing-Long-Tang | Minor Green-Blue Dragon Decoction | 949 | Ma-Huang(Ephedrae Herba Cruda), Gui-Zhi(cinnamon twig Cinnamomi Ramulus), Bai-Shao(white peony [root] Paeoniae Radix Alba), Gan-Cao(Glycyrrhizae Radix cum Liquido Fricta), Gan-Jiang(dried ginger Zingiberis Rhizoma), Xi-Xin(as arum Asari Herba), Ban-Xia(pinellia [rhizome] Pinelliae Rhizoma), Wu-Wei-Zi(schisandra [berry] Schisandrae Fructus) |
| 半夏厚朴湯 | Ban-Xia-Hou-Po-Tang | Pinellia and Officinal Magnolia Bark Decoction | 836 | Ban-Xia(pinellia [rhizome] Pinelliae Rhizoma), Hou-Po(officinal magnolia bark Magnoliae Officinalis Cortex), Fu-Ling(Poria cum Cute), Gan-Jiang(dried ginger Zingiberis Rhizoma), Su-Ye(perilla leaf Perillae Folium) |
| 辛夷清肺湯 | Xin-Yi-Qing-Fei-Tang | Magnolia Flower Lung-Clearing Decoction | 805 | Xin-Yi(magnolia flower Magnoliae Flos), Bai-He(lily bulb Lilii Bulbus), Zhi-Mu(anemarrhena [root] Anemarrhenae Rhizoma), Shi-Gao(Gypsum Crudum), Pi-Pa-Ye(loquat leaf Eriobotryae Folium), Sheng-Ma(cimicifuga [root] Cimicifugae Rhizoma), Mai-Dong(ophiopogon [root] Ophiopogonis Radix), Zhi-Zi(gardenia [fruit] Gardeniae Fructus), Huang-Qin(scutellaria [root] Scutellariae Radix), Gan-Cao(Glycyrrhizae Radix cum Liquido Fricta) |
| 杏蘇散 | Xing-Su-San | Apricot Kernel and Perilla Powder | 789 | Su-Ye(perilla leaf Perillae Folium), Jie-Geng (platycodon [root] Platycodonis Radix), Da-Zao(jujube Jujubae Fructus), Ban-Xia(pinellia [rhizome] Pinelliae Rhizoma), Zhi-Qiao(bitter orange Aurantii Fructus), Ju-Pi (tangerine peel Citri Reticulatae Pericarpium), Fu-Ling(Poria cum Cute), Gan-Cao(Glycyrrhizae Radix cum Liquido Fricta), Xing-Ren(Armeniacae Semen), Qian-Hu(peucedanum [root] Peucedani Radix), Gan-Jiang(dried ginger Zingiberis Rhizoma) |
| 辛夷散 | Xin-Yi-San | Magnolia Flower Powder | 769 | Xin-Yi(magnolia flower Magnoliae Flos), Bai-Zhi(Dahurian angelica Angelicae Dahuricae Radix), Sheng-Ma(cimicifuga [root] Cimicifugae Rhizoma), Gao-Ben (Chinese lovage [root] Ligustici Rhizoma), Fang-Feng (Ligustici Brachylobi Radix), Chuan-Xiong(chuanxiong [rhizome] Chuanxiong Rhizoma), Xi-Xin(as arum Asari Herba), Mu-Tong(Armand's clematis [stem] Clematidis Armandii Caulis), Gan-Cao(Glycyrrhizae Radix cum Liquido Fricta) |
| 天王補心丹 | Tian-Wang-Bu-Xin-Dan | Celestial Emperor Heart-Supplementing Elixir | 763 | Tian-Dong(asparagus [root] Asparagi Radix), Ren-Shen(ginseng Ginseng Radix), Fu-Ling(Poria cum Cute), Xuan-Shen(scrophularia [root] Scrophulariae Radix), Dan-Shen(salvia [root] Salviae Miltiorrhizae Radix), Yuan-Zhi(polygala [root] Polygalae Radix), Jie-Geng (platycodon [root] Platycodonis Radix), Dang-Gui(tangkuei [root] Angelicae Sinensis Radix Integra), Wu-Wei-Zi(schisandra [berry] Schisandrae Fructus), Mai-Dong(ophiopogon [root] Ophiopogonis Radix, Bai-Zi-Ren (arborvitae seed Platycladi Semen), Suan-Zao-Ren(spiny jujube [kernel] Ziziphi Spinosi Semen), Sheng-Di-Huang(rehmannia [root] Rehmanniae Radix (Exsiccata seu Recens)) |
| 川芎茶調散 | Chuan-Xiong-Cha-Tiao-San | Tea-Blended Chuanxiong Powder | 665 | Bo-He(mint Menthae Herba), Fang-Feng (Ligustici Brachylobi Radix), Xi-Xin(as arum Asari Herba), Qiang-Huo (notopterygium [root] Notopterygii Rhizoma et Radix), Bai-Zhi(Dahurian angelica Angelicae Dahuricae Radix), Gan-Cao(Glycyrrhizae Radix cum Liquido Fricta), Chuan-Xiong(chuanxiong [rhizome] Chuanxiong Rhizoma), Jing-Jie-Sui(schizonepeta spike Schizonepetae Flos) |
| 止嗽散 | Zhi-Sou-San | Cough-Stopping Powder | 644 | Jie-Geng (platycodon [root] Platycodonis Radix), Jing-Jie-Sui(schizonepeta spike Schizonepetae Flos), Zi-Wan (aster [root] Asteris Radix), Bai-Bu(stemona [root] Stemonae Radix), Bai-Qian(willowleaf swallowwort [rhizome] Cynanchi Stauntonii Rhizoma), Gan-Cao(Glycyrrhizae Radix cum Liquido Fricta), Chen-Pi(tangerine peel Citri Reticulatae Pericarpium) |
